# Retreatment of recurrent adult medulloblastoma with radiotherapy: a case report and review of the literature

**DOI:** 10.1186/1752-1947-7-64

**Published:** 2013-03-08

**Authors:** Michela Buglione, Luca Triggiani, Salvatore Grisanti, Roberto Liserre, Luciano Buttolo, Stefano Gipponi, Fausta Bonetti, Alice Todeschini, Luigi Spiazzi, Stefano Maria Magrini

**Affiliations:** 1 Radiation Oncology Department, Brescia University, P.le Spedali Civili 1, 25123 Brescia, Italy; 2 Medical Oncology Department, Spedali Civili Hospital, Brescia, P.le Spedali Civili 1, 25123 Brescia, Italy; 3 Neuroradiology Department, Brescia University, P.le Spedali Civili 1, 25123 Brescia, Italy; 4 Neurosurgery Department, Brescia University, P.le Spedali Civili 1, 25123 Brescia, Italy; 5 Neurology Department, Brescia University, P.le Spedali Civili 1, 25123 Brescia, Italy; 6 Pathology Department, Brescia University, P.le Spedali Civili 1, 25123 Brescia, Italy; 7 Physics Department, Spedali Civili Hospital, Brescia, P.le Spedali Civili 1, 25123 Brescia, Italy

**Keywords:** Adult medulloblastoma, Biological equivalent dose, Re-irradiation

## Abstract

**Introduction:**

Medulloblastoma, the most frequent brain tumor in childhood, also occurs with a wide range of characteristics in adult patients. Late relapse is common in adult medulloblastoma, and the overall survival of relapsed patients usually ranges from 12 to 15 months. Treatment at recurrence is still debated and after reoperation includes stereotactic or normofractionated radiotherapy, and high-dose chemotherapy with autologous bone marrow transplantation.

**Case presentation:**

We report on the case of a 31-year-old Caucasian woman who underwent re-irradiation for a recurrence of medulloblastoma at nine years after first irradiation (56Gy), focusing on the radiobiological background and a review of previous studies involving re-irradiation of recurrent medulloblastoma. After surgical excision of the relapsed tumor and medical multi-agent treatment, the site of recurrence was treated using three-dimensional conformal radiotherapy to a total dose of 52.8Gy (1.2Gy/fraction/twice daily). A total biological equivalent dose of 224.6Gy (α:β = 2 Gy) was delivered to the posterior fossa (first and second treatments). No radionecrosis or local recurrence was evident at 18 months after re-irradiation.

**Conclusion:**

Re-irradiation can be considered a possible and safe treatment in selected cases of recurrent medulloblastoma in adults. The reported radiobiological considerations could be useful in other cases involving re-irradiation of brain tumors.

## Introduction

Medulloblastoma is a primitive neuro-ectodermal tumor that is the most frequent primary brain neoplasm in childhood. About 50% of these tumors occur in children aged less than five years, whereas they are rare in adolescents and young adults. Medulloblastoma behaves differently in adults than in children, and is identified as a different biological and clinical entity [1]. It exhibits a higher proportion of desmoplastic histological characteristics than childhood medulloblastoma and shows a higher incidence within the cerebellar hemispheres, thus featuring different proliferative and apoptotic indices and having a tendency for late relapse [2]. The application of pediatric strategies in the management of this tumor in adults has shown some success. As the data in the literature are based on retrospective studies, treatments have been neither randomized nor uniform. However, some treatment cornerstones have been identified. The first step is surgical excision carried out as completely as possible without resulting in major neurological impairment. The second step is radiotherapy. A total dose of at least 55Gy to the posterior fossa and 36Gy to the remaining cranial–spinal axis is needed; adjuvant chemotherapy might be useful in patients at high risk of recurrence (Chang’s classification) especially if distant metastases are present [3]. In adult patients, the five-year progression-free survival rate ranges from 45% to 75% depending on the risk class [2,3].

The outcome for patients with recurrent medulloblastoma has historically been poor, with most patients dying of disseminated disease: usually within 12 to 15 months after retreatment with either chemotherapy or radiotherapy. The optimal treatment for local relapse in non-metastatic patients remains controversial. A number of chemotherapy regimens (with or without high-dose chemotherapy and bone marrow autologous transplantation) have been proposed, along with temozolomide [4-6] and single-fraction stereotactic radio-surgery [7] or stereotactic hypo-fractionated radiotherapy [8]. Re-irradiation is frequently undertaken for isolated brain relapses. A meta-analysis of brain re-irradiation found no cases of necrosis if the total dose was lower than 100Gy (two Gy/fraction; α:β = 2 Gy). A full dose re-irradiation can be considered an option; it depends on the time elapsed since primary treatment and on the feasibility of a surgical resection of the recurrence [9].

## Case presentation

The patient, a 31-year-old woman, complained of headache and dizziness and underwent magnetic resonance imaging (MRI) of the brain; this showed a contrast-enhancing lesion in the posterior fossa. Radical removal of the lesion was achieved by means of right posterior fossa craniectomy and laminectomy of the first cervical vertebra; histological examination confirmed the diagnosis of T2 (tumor greater than three cm and invading one adjacent structure or partially filling fourth ventricle) M0 (no gross subarachnoid or hematogenous metastasis) (Chang’s classification), desmoplastic medulloblastoma. At one month after surgery, cranio-spinal irradiation was carried out. The entire cranio–spinal axis was treated. The treatment consisted of two parallel opposed photon fields, latero–lateral for the brain and posterior–anterior for the spinal axis, using the moving junction technique. The total dose to the brain and spinal axis was 36Gy in 20 fractions (1.8Gy/fraction; five fractions/week; one fraction/day). A boost to the posterior cranial fossa (18Gy in 10 fractions) was then delivered, resulting in a total dose to the tumor bed of 54Gy. Radiotherapy was well tolerated and the patient did not show any acute toxicity, with the exception of mild neutropenia and nausea.

The patient underwent regular follow-up at the Radiation Oncology Department without evidence of recurrent disease. Eight years later, MRI showed a recurrence in the right cerebellum (cranio-caudal diameter, 5.7cm; antero-posterior diameter, 4.7cm), causing a bulge in the IV ventricle. Caudally the lesion imprinted the right cerebellar tonsil, resulting in a reduction in the ipsilateral foramen of Luschka. The patient was in good general condition; neurological examination did not reveal any neurological deficit. Neurocognitive analysis obtained by submitting the patient to a standardized neurological examination using the Mini-Mental® State Examination (MMSE), Clock Drawing Test, Rey auditory verbal learning test and Trail Making Test (TMT) indicated a normal global cognitive function (MMSE, 28 out of 30), with important disexecutive and attention deficits (Clock Drawing Test, three out of 10 and mild pathological TMT). No memory impairment was detected using the MMSE and Rey Auditory-Verbal Learning Tests.

**Figure 1:**
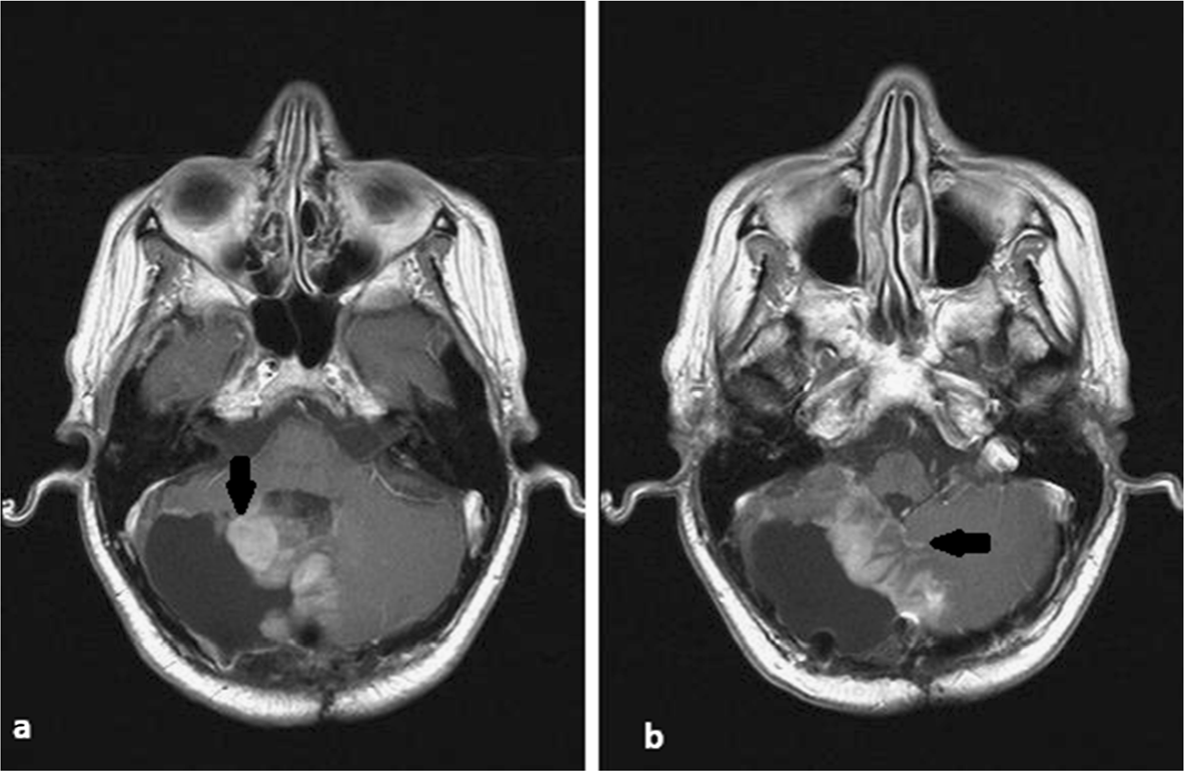
**a and b: The magnetic resonance imaging before re-irradiation.** Recurrence of disease is clearly evident (*arrows*).

After multidisciplinary clinical discussion the patient underwent partial surgical resection of the recurrence. The post-surgical MRI evidenced a small nodule on the edge of the superior-medial cavity, showing post-contrast enhancement that was suggestive of residual disease. The patient then received salvage chemotherapy including carboplatin, procarbazine, etoposide, cisplatin, vincristine and cyclophosphamide according to the sequential trial of the French Society of Paediatric Oncology [10]. After an initial response to treatment, the patient refused high-dose chemotherapy with autologous stem cell support. She continued with the same schedule for about one year, until the disease progressed at the margins of the surgical cavity, vermis and right cerebellar hemisphere. Second line palliative chemotherapy with low-dose continuous temozolomide was administered for three months without response. Brain, brainstem and spinal MRI showed a marked increase in the number and size of the nodules present in the posterior fossa (Figure 1 a,b). Given the occurrence of local progression, the time that had elapsed from initial radiotherapy (nine years) and the biological efficacy dose (BED) of the first treatment *(*α:β = 2 Gy; 1.8Gy/fraction in 30 fractions; BED = 102.6Gy), a salvage re-irradiation was considered.

**Figure 2:**
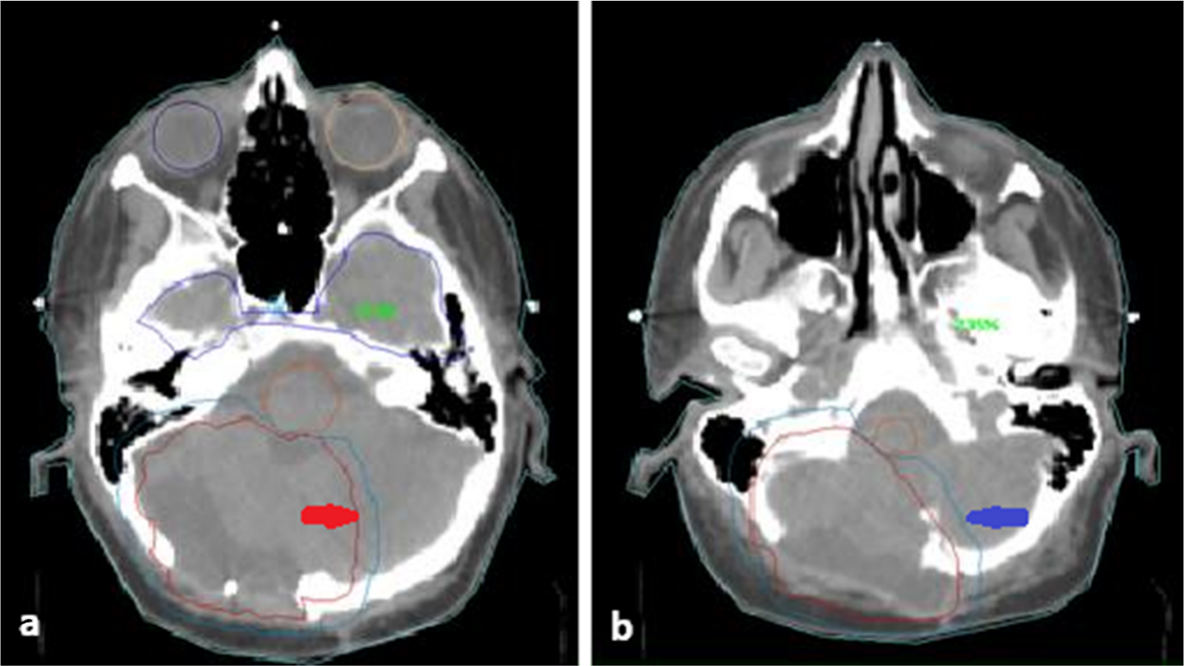
**a and b: Planning computed tomography with the retreatment gross tumor volume/clinical target volume and planning target volume identified in red (*red arrow*) and in blue (*blue arrow*), respectively.**

The patient signed an informed consent declaration, after being informed of the possible acute and late toxicities related to the re-irradiation. With the patient in the supine treatment position and immobilized with a thermoplastic mask, we obtained whole brain computed tomography (CT) with three-mm slices from the vault of the skull to C5. The volumes were defined on the planning CT and were co-registered and fused with the diagnostic MRI images (Figure 2 a,b). The planning target volume consisted of the five-mm expansion of the clinical target volume, encompassing the gross tumor volume and surgical cavity. Even if the total dose delivered to the spinal liquoral spaces during the first line treatment was lower than that to the posterior fossa, the cord was not included in the treatment volume because it was free of disease. The patient received three-dimensional conformal radiotherapy to a total dose of 52.8Gy in 44 fractions over 22 days (1.2Gy/fraction; two fractions daily; 10 fractions weekly; α:β = 2 Gy; BED=122Gy).

**Figure 3:**
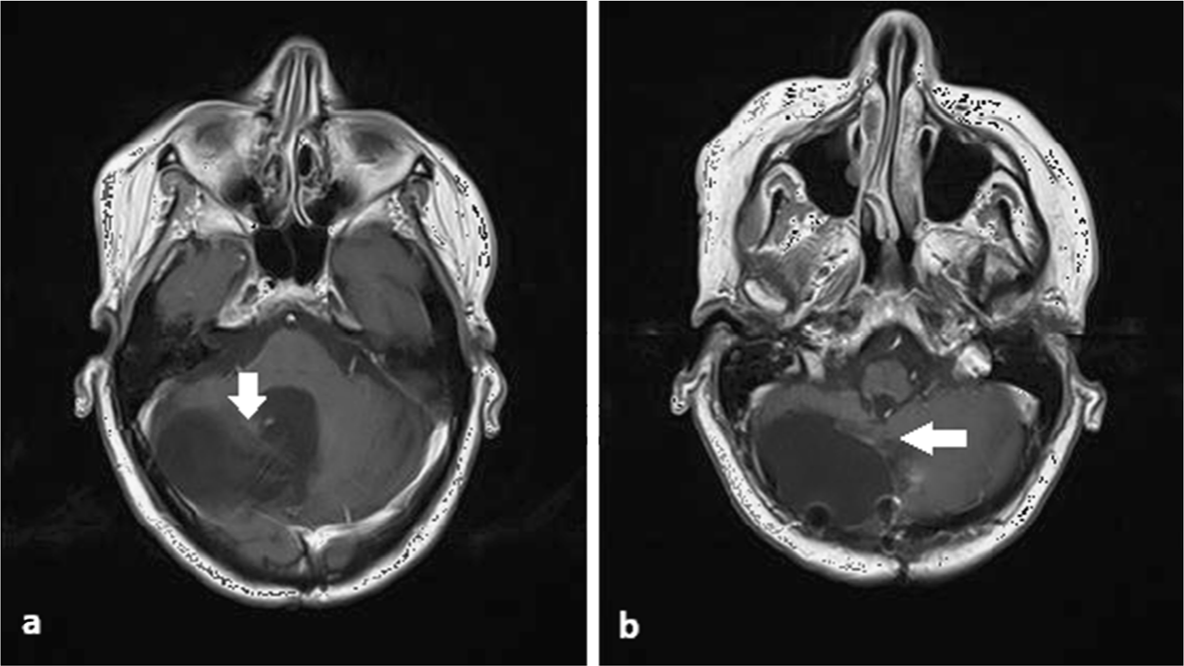
**a and b: The magnetic resonance imaging performed at three months after re-irradiation showed complete remission (*white arrows*).**

**Figure 4:**
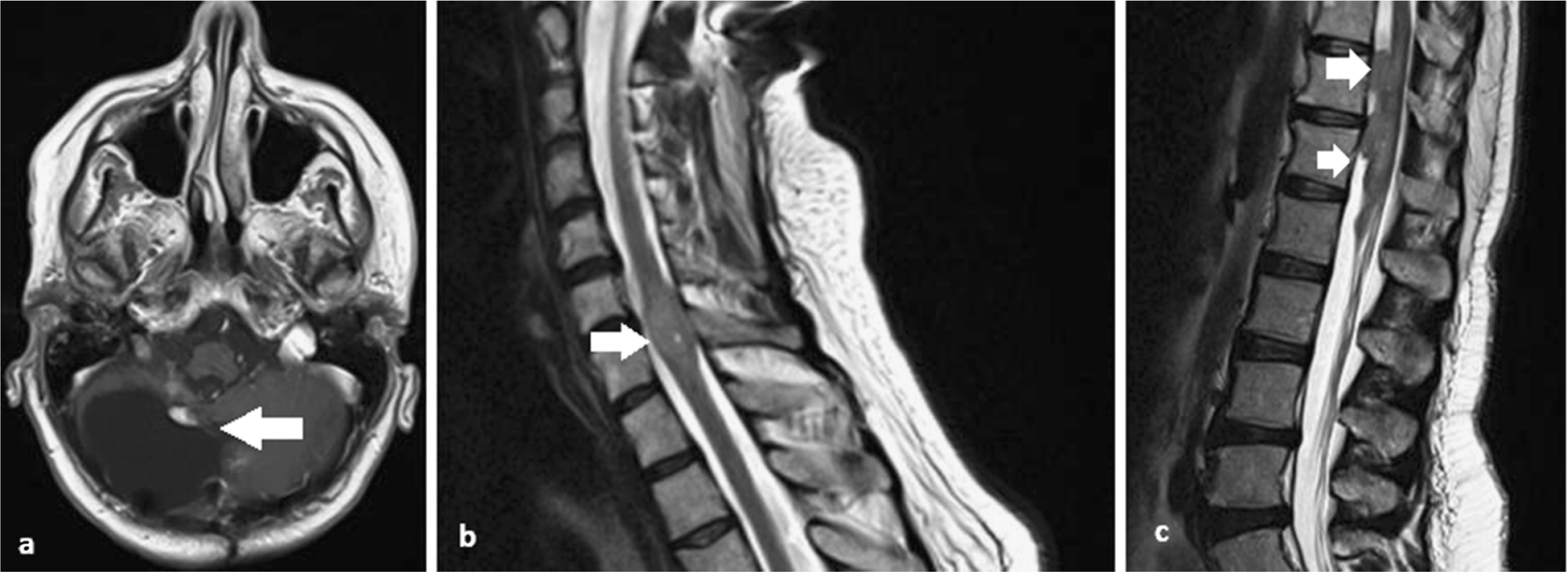
**a, b and c: The magnetic resonance imaging performed at one year after re-irradiation showed disease recurrence in the spine and no brain toxicity (*white arrows*).**

The treatment was well tolerated and the acute and sub-acute toxicities were mild; slight somnolence and asthenia disappeared within two months from the end of the treatment. The patient continued with follow-up at our institution. MRI, obtained at three months after re-irradiation, showed complete regression of the multiple nodules in the posterior fossa and no evidence of radionecrosis (Figure 3 a,b). Unfortunately, at 10 months after re-irradiation, the patient developed paraplegia. MRI revealed local relapse and multiple sites of spinal recurrence of disease, which were responsible for the reported symptoms (Figure 4 a,b,c). Despite the fact that a radical re-irradiation of the entire spine with a boost delivered to macroscopic disease was considered, a palliative treatment was chosen to treat only the sites of recurrence (30Gy in 10 fractions). At 18 months after re-irradiation, no radionecrosis or other late toxicities were evident within the re-treated area on MRI.

## Discussion

The risk of toxicity can differ between brain regions with a highly collateralized blood supply and those perfused by only a few small branches [11]. Importantly, and in contrast to the spinal cord and other organs, few animal studies regarding re-irradiation of the brain have been published. Late injury is pathologically characterized by demyelination, vascular changes and ultimately necrosis [10]. Therefore, much attention has been paid to the radiation response of the vasculature and the oligodendrocyte population. Radiation induces damage in endothelial cells and the loss of 02A progenitor cells; this results in failure to replace oligodendrocytes and subsequent demyelination [12]. However, the biology of late central nervous system toxicity is thought to be a complex dynamic process, possibly involving other cell types (astrocytes, microglia, neurons and neural stem cells) and different cell interactions. No effective means of prevention or treatment are known [10,12]. The response of brain tissue to irradiation seems similar to that of the spinal cord. Consequently, most of the available data are extrapolated from studies on the spinal cord of rhesus monkeys [13]. In humans, there is evidence that the risk of myelopathy is low at radiation doses of up to a median cumulative BED of 135Gy (α:β = 2 Gy for cervical and thoracic cord and 4 Gy for lumbar cord), when the time interval between re-irradiation is not shorter than six months and the dose for each course is <98Gy BED [14]. Data exist concerning the re-irradiation of brain tumors to a median cumulative BED_2_ (biological equivalent dose in 2Gy fractions) of 200Gy, with at least one year between the two treatments; long-term complications related to the retreatment were seen in patients with a BED_2_>204Gy (α:β = 2 Gy) [15].

In the largest published medulloblastoma re-irradiation series (13 patients), the dose prescribed for the second treatment was 30Gy (1.5Gy per fraction); the median cumulative dose reached 82.3Gy (range 73.8 to 98.4Gy) [10]. In this series, the treatment volumes included the site of recurrence, and the spine was only re-irradiated if it was the site of recurrence. Cranio-spinal irradiation was carried out in just one of the cases because of the time that had elapsed from the first treatment (eight years).

The fractionation regimen (52.8Gy in 44 fractions; 1.2Gy/fraction; two fractions daily; 10 fractions weekly) was chosen to take advantage of the early-repair characteristics of medulloblastoma cells, as opposed to the late repair of healthy brain tissue. The theoretical cumulative BEDs for late and early effects on the healthy tissue of the posterior fossa (healthy brain) were of 74.3Gy and 78Gy, respectively (Additional file 1). The initial treatment involved a tumor BED of 102.6Gy, a late effects BED of 86.4Gy and an early effects BED of 63.7Gy. Therefore, the overall treatment involved a tumor BED of 224.6Gy, a late reaction BED of 160.7Gy and an early reaction BED of 141.7Gy, without taking into account the recovery due to the gap between the first and the second treatment. The total physical dose to the brainstem was limited to 49Gy.

## Conclusions

In our experience, re-irradiation of recurrent medulloblastoma is feasible and safe without evidence of acute or late toxicity after 18 months of follow-up. Even if radionecrosis can occur later than 18 months, this follow-up period can be considered a sufficient time interval because a significant part of the slow repair process occurs within one year. A radical dose (60Gy) can be delivered, especially if given at low doses per fraction. The spinal cord can possibly be included in the treatment volume, even if it is not involved with the recurrence, to avoid early spinal progression.

Because of concerns with regard to the risk of late central nervous system toxicity, especially radionecrosis that may occur several months to years after treatment, radiation oncologists have historically been cautious about retreating brain tumors. However, there is a lack of prospective data in this regard. Re-irradiation of brain tumors is attracting increasing interest. In fact, a greater understanding of brain tolerance to radiation, developments in tumor imaging and advances in radiotherapy planning and delivery techniques now make possible the achievement of better target definition and highly conformal treatments.

## Consent

Written informed consent was obtained from the patient for publication of this case report and any accompanying images. A copy of the written consent is available for review by the Editor-in-Chief of this journal.

## Abbreviations

BED: Biological efficacy dose; CT: Computed tomography; MMSE: Mini-Mental® State Examination; TMT: Trail Making Test.

## Competing interests

The authors declare that they have no competing interests.

## Authors’ contributions

MB treated the patient with radiotherapy, analyzed and interpreted the data, coordinated all of the authors, and ideated and drafted the article. LT treated the patient with radiotherapy and drafted the article. SG treated the patient with chemotherapy and drafted the article. RL performed the magnetic resonance imaging and drafted the article. LB surgically treated the patient and drafted the article. SG performed neurologic evaluations and drafted the article. FB performed pathological evaluation. AT performed neurologic evaluations. LS carried out dosimetric evaluation and physical treatment planning, and drafted the article. SMM was responsible for patient treatment, analyzed and interpreted the data, revised the article and approved the final version. All authors read and approved the final manuscript.

## Authors’ information

All of the authors are members of the Neuro-Oncology Multidisciplinary Group, Spedali Civili Hospital and Brescia University, Brescia, Italy.

## Supplementary Material

Additional file 1 Appendix. Click here for file
