# Supplementary material for: Retreatment of recurrent adult medulloblastoma with radiotherapy: a case report and review of the literature
Source: J Med Case Rep. 2013 Mar 8;7:64. doi: 10.1186/1752-1947-7-64 (PMC3599802; doi:10.1186/1752-1947-7-64)
Supplement: Additional file 1 — Appendix. [file 1752-1947-7-64-S1.doc]

**Additional file 1**

In fractionated radiotherapy, the biologically effective dose (BED) is the total dose required to produce a particular effect in different tissues and organs. Each tissue is characterized by a different level of sensitivity to acute or late damage, which is expressed by the ratio of the parameters *α* and *β.* The *α* parameter, which is typical of tissues with a high proliferative index, expresses sensitivity to acute radiation damage. The *β* parameter*,* which is typical of tissues with a low proliferative index, expresses sensitivity to late radiation damage. The *α*/*β* ratio is used within a mathematical model (the “linear quadratic model”) to explain the toxicity related to ionizing radiation. The tissues that are more sensitive to acute damage are characterized by a high *α*/*β* ratio (for example bone marrow and mucosa), whereas those with a low *α*/*β* ratio are more sensitive to late damage (for example central nervous system).

Two corrections have been applied to evaluate the appropriate BED for the chosen dose schedule; the first one takes into account the short recovery time for the bi-daily strategy; the second one takes into account the reduced overall time as compared with a single daily fraction scheme.

1. Incomplete repair

Assuming that for the type of tumor evaluated in the present study the recovery half time is five hours and that the time gap between the fractions is seven hours, the incomplete repair factor Hm is 0.4 (Table 8.2 in [1]).

Therefore the provisional tumor equivalent dose in 2Gy fractions (EQD_2_) is:

EQD_2. bi-daily_=52.8Gy × [1.2 × (1 + 0.4 ) + 2 ]/(2 + 2)=48.6Gy

This represents a 15% increase in efficacy as compared with the uncorrected value (42.2Gy).

It is important to take into account that not all the fractions were delivered with a time gap of seven hours. Indeed, the time gap was seven hours for the fractions delivered in the same day, and 17 hours for the second fraction on day A and the first fraction on day A+1. Therefore, it seems more appropriate to consider an incomplete repair effect that is half of the estimated one (a 7.5 % increase) that brings the tumor EQD_2_ to:

EQD_2, bi-daily_=45.4Gy

1. Shortened overall treatment time

Considering a single fraction per day and a five day per week schedule, 44 fractions would be delivered in 60 days (eight weeks + four days), whereas for a bi-daily schedule the overall treatment time would be 30 days (four weeks + two days). Using a value of dose recovered per day owing to proliferation of 0.52Gy (Table 9.3 in [1]), reducing the overall treatment time from 60 days to 30 days would cause an increase in EQD_2_ of:

EQD_2_ = (60–30) × 0.52Gy=15.6Gy

Globally, the EQD_2_ (1.2Gy × 44 fractions; bi-daily; α/β= two Gy) is therefore:

EQD_2; 1,2bid_=45.4Gy + 15.6Gy=61Gy

This corresponds to a BED (α/β= two Gy)=122Gy.

Furthermore, the choice of a bi-daily schedule is directed to increase the therapeutic gain between normal tissue and tumor. The incomplete repair effect regarding normal tissues surrounding the tumor would, in fact, be lower than that for medulloblastoma. Considering the brain the critical structure, we can use the same calculations assuming an α/β= three Gy and 10Gy for late reactions and early reactions, respectively, and a recovery half time of 1.5 hours (Table 8.4 in [1]).

1. Late reactions

EQD_2_, bi-daily,OAR (organs at risk)=52.8Gy × [1.2 × (1 + 0.045 ) + 3 ]/(2 + 3)=44.9Gy

This represents a 1.3% increase in efficacy relative to the uncorrected value (44.3Gy).

Therefore, considering a corrected increase in efficacy of 0.7% gives:

EQD_2, bi-daily,OAR_=44.6Gy

1. Early reactions

EQD_2, bi-daily,OAR_=52.8Gy × [1.2 × (1 + 0.045 ) + 10 ]/(2 + 10)=49.5Gy

This represents a 0.4% increase in efficacy as compared with the uncorrected value (49.3Gy).

Considering a corrected increase in efficacy of 0.2% gives:

EQD_2, bi-daily,OAR_=49.4Gy

The effect of shortened overall treatment time can be neglected in relation to late effects, whereas for early reactions we can assume an effect similar to the one for tumor. Therefore:

Late effects: EQD_2, bi-daily,OAR_=44.6Gy (BED=74.3Gy)

Early effects: EQD_2, bi-daily,OAR_=65.0Gy (BED=78Gy)

A standard fractionation of 2.4Gy for 22 fractions would then result in:

Tumor:

EQD_2_ (2.4Gy/fraction; 22 fractions; α/β= two Gy) = 58.1Gy (95% of the one for the chosen schedule).

Brain late effects:

EQD_2_ (2.4Gy/fraction; 22 fractions; α/β= three Gy)=57.0Gy (128% of the one for the chosen schedule).

EQD_2_ (2.4Gy/fraction; 22 fractions; α/β=10Gy)=65.5Gy (100.8% of the one for the chosen schedule).

**Reference**

1. Joiner M.A.C. and Bentzen S.M.: Fractionation: the linear quadratic approach; The linear-quadratic approach in clinical practice . In Basic Clinical Radiobiology 4th edition. Edited by Edward Arnold: Joiner M. and van der Kogel A. 2009: 102-134.
